# Pathogenesis and seroprevalence of dengue virus in HIV-endemic regions: A global perspective

**DOI:** 10.1016/j.nmni.2025.101672

**Published:** 2025-11-18

**Authors:** Blessed T. Mukuhlani

**Affiliations:** University of Zimbabwe, Zimbabwe

**Keywords:** Dengue virus, HIV coinfection, Immunopathogenesis, Arboviral surveillance, Diagnostic biosensors, Seroprevalence

## Abstract

Dengue virus (DENV), a globally prevalent flavivirus, is a growing threat in regions where HIV is endemic. The co-endemicity of HIV and dengue poses overlapping clinical challenges, particularly in low-resource settings where diagnostic capacity is limited. This review examines the epidemiological overlap, immunological interactions, and diagnostic difficulties associated with DENV infection in people living with HIV (PLWH). Seroprevalence studies reveal high rates of dengue exposure among PLWH in sub-Saharan Africa, Latin America, and Southeast Asia, with increasing evidence of autochthonous transmission in non-endemic regions. Immunologically, coinfection is characterized by altered platelet function, suppressed chemokine secretion, and dysregulated T cell activation. Molecules such as CLEC5A and CD300a play key roles in the immune modulation seen in coinfected hosts. Although clinical presentations vary from mild to severe, atypical manifestations including encephalitis and hepatic dysfunction have been observed. Diagnostic confusion with acute HIV syndrome or other febrile illnesses remains a major concern. Innovations such as CRISPR-based detection systems and graphene biosensors hold promise for field-deployable diagnostics. Mathematical modeling supports integrated control strategies, and novel DENV-derived peptides like pepRF1 show potential as dual-acting therapeutics. As climate change facilitates the spread of Aedes vectors, dengue's expansion into new territories highlights the urgency of integrating arboviral surveillance with HIV care systems. This review underscores the need for enhanced diagnostics, better therapeutic options, and longitudinal research on DENV pathogenesis in immunocompromised populations.

## 1 Introduction

**Fig. 1:**
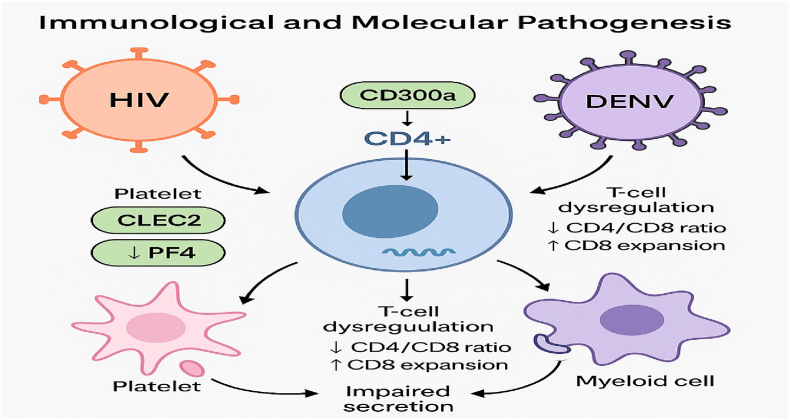
Immunological and molecular pathogenesis of HIV–dengue virus (DENV) coinfection.

Dengue virus (DENV), a mosquito-borne flavivirus, continues to pose a significant global public health challenge. The increasing incidence and geographic expansion of dengue are largely attributed to urbanization, global travel, and climate change, which have facilitated the spread of mosquito vectors such as Aedes aegypti and Aedes albopictus [1,2,3]. Dengue is endemic in more than 100 countries and causes an estimated 390 million infections annually, with nearly 96 million manifesting clinically. Concurrently, human immunodeficiency virus (HIV) remains a major burden in low- and middle-income countries. As a result, the co-endemicity of dengue and HIV has become increasingly relevant, particularly in resource-limited settings where both viruses co-circulate (see Fig. 1).

Coinfection with dengue and HIV presents a unique set of clinical and diagnostic challenges. The immunosuppressed state of people living with HIV (PLWH) may affect the clinical presentation and severity of dengue, potentially leading to atypical manifestations or exacerbation of disease outcomes [4,5]. Moreover, misdiagnosis is common due to overlapping symptoms such as fever, fatigue, rash, and gastrointestinal disturbances. Reports of dengue in HIV-positive individuals have emerged from endemic and non-endemic regions alike, underscoring the global implications of these interactions [6,7].

This review synthesizes the latest evidence on the epidemiology, clinical manifestations, immunological mechanisms, diagnostic challenges, and public health implications of dengue virus infection, particularly in the context of HIV coinfection. The analysis focuses exclusively on DENV, providing a comprehensive understanding of its impact among immunocompromised populations. Unlike previous reviews that addressed either dengue or HIV in isolation, this paper provides the first comprehensive synthesis linking immunopathogenesis, epidemiology, diagnostics, and emerging therapeutic strategies in the context of HIV–dengue coinfection, with an explicit focus on sub-Saharan Africa, where overlapping transmission and underreporting complicate surveillance. By consolidating both clinical and molecular perspectives, this work contributes a multi-level framework to guide diagnostic policy, research prioritization, and coinfection management in co-endemic regions.

## 2 Methodology

This narrative review was conducted according to the SANRA (Scale for the Assessment of Narrative Review Articles) criteria to ensure transparency, balanced referencing, and a clear conceptual framework. A comprehensive literature search was performed across PubMed, Embase, Scopus, Web of Science, and African Journals Online (AJOL) covering the period from January 1, 2000, to October 10, 2025. The search used combinations of the keywords “dengue,” “dengue virus,” “DENV,” “HIV,” “human immunodeficiency virus,” and “coinfection.” Additional grey literature and reference lists of retrieved studies were also screened for relevant data. Only English-language publications reporting dengue virus infection in people living with HIV (PLWH) were included, with particular focus on evidence from sub-Saharan Africa. Data were extracted and synthesized narratively, emphasizing epidemiology, immunopathogenesis, clinical manifestations, diagnostics, and coinfection patterns. The review critically appraised the methodological quality and contextual validity of included studies, providing a reproducible and comprehensive synthesis consistent with high-quality narrative review standards.

## 3 Epidemiology of Dengue–HIV coinfection

Epidemiological studies reveal a concerning overlap between regions heavily affected by HIV and those experiencing dengue outbreaks. In sub-Saharan Africa and parts of Latin America, coinfections are frequently underreported due to limited testing and overlapping symptomatology. For instance, a Nigerian study found that 44.4 % of febrile HIV-infected patients were seropositive for dengue virus IgG [8]. In coastal Kenya, 8.8 % of febrile adults screened for acute HIV infection and malaria tested positive for DENV [9]. Similarly, in Madagascar, high seroprevalence for DENV-1 (12.4 %) and other arboviruses has been documented in PLWH, with regional variations correlating to rainfall patterns [10].

In Mexico, HIV-dengue coinfections have been reported with clinical evidence of dengue virus serotype 1 among patients receiving antiretroviral therapy (ART), where coinfection did not appear to significantly affect HIV viral load or CD4 counts [6]. Emerging evidence from non-endemic regions further underscores the global relevance of coinfection. During the 2023 dengue outbreak in Italy, seroprevalence among PLWH was 7.8 %, with some patients seroconverting despite no travel history—confirming local transmission [7]. These data highlight the potential for dengue to spread into previously unaffected areas and the importance of considering dengue in febrile illness among HIV patients globally [11].

## 4 Clinical presentations and case reports

The clinical spectrum of dengue in PLWH varies widely. While some studies report milder dengue symptoms in PLWH, others describe atypical or severe manifestations. A Brazilian cohort found reduced inflammation and lower risk of vascular instability in HIV-dengue coinfected patients, likely due to platelet dysfunction and chemokine suppression (RANTES, PF4) [12]. Another study confirmed increased CD8^+^ T cell populations and altered T cell activation markers (CCR5, CD107a) in coinfected patients [13].

More severe outcomes have been documented as well. A case of dengue virus encephalitis in an Australian HIV-positive woman indicated potential chronic neurotropism, with active DENV detected in brain tissue months after exposure [14]. Coinfections involving hepatitis B virus (HBV) and dengue in HIV-positive individuals have also been reported, with hepatic enzyme elevation during infection [15].

Collectively, these cases demonstrate that while some PLWH may experience attenuated dengue illness due to immune suppression, others may suffer from serious complications, highlighting the heterogeneity of disease outcomes in coinfection [4,5,6].

In this review, past dengue virus exposure refers to the detection of anti-DENV IgG antibodies, while acute or recent infection denotes detection of NS1 antigen, RT-PCR positivity, or IgM seroconversion. Interpretation of serological findings must consider the extensive cross-reactivity between dengue and other flaviviruses such as Zika virus, yellow fever, and West Nile virus, particularly in regions with overlapping transmission or routine yellow-fever vaccination. Consequently, plaque-reduction neutralization testing (PRNT) or virus-specific neutralization assays remain the reference standard for confirmation in co-endemic areas. Many seroprevalence studies in Africa and Latin America did not perform PRNT, which may lead to over-estimation of dengue exposure. This limitation is acknowledged in the synthesis of epidemiological data.

## 5 Immunological and molecular pathogenesis

HIV alters host immunity in ways that can influence dengue pathogenesis. Platelets, which act as immune mediators, show impaired secretion of inflammatory chemokines like RANTES and PF4 in coinfected individuals, correlating with reduced inflammatory response and potentially milder clinical manifestations [12]. Simultaneously, CD8^+^ T cell expansion and abnormal activation marker expression suggest a complex interplay between viral persistence and immune modulation [13].

Receptors such as CLEC2 and CLEC5A, expressed on platelets and myeloid cells respectively, have emerged as important mediators in viral-induced NETosis and cytokine storms. Activation of these receptors by DENV can exacerbate inflammatory responses, especially in immunocompromised hosts [16]. Moreover, CD300a, a molecule overexpressed on memory CD4^+^ T cells in HIV patients, has been associated with increased susceptibility to both HIV and dengue infections, underscoring the immunological overlap between these viruses [17]. Consistent with cytokine-driven vascular pathology, a prospective Indonesian study showed higher TNF-α, IL-6, and IL-17 in DHF versus DF, while IL-10 was higher in DF; secondary dengue also had higher TNF-α, IL-6, IL-17 and lower IL-10 than primary infection, aligning with a more pro-inflammatory milieu and plasma-leak risk. These patterns support our thesis that immune skewing modulates severity and may interact with HIV-related immune dysregulation in PLWH. “Recent studies further demonstrate that HIV-related immune dysregulation amplifies dengue-induced cytokine activation and interferon signaling, leading to more severe inflammatory profiles in coinfected hosts [18].

Schematic representation showing the key immune pathways affected during HIV–DENV coinfection. HIV infection alters platelet signaling through CLEC2 and decreases PF4 secretion, while DENV activates platelets and myeloid cells via pattern-recognition receptors such as CLEC5A. Both viruses contribute to T-cell dysregulation, characterized by a reduced CD4/CD8 ratio and expansion of CD8^+^ T cells. The inhibitory receptor CD300a, upregulated on CD4^+^ T cells, enhances viral susceptibility and immune imbalance. Together, these mechanisms lead to impaired immune secretion, altered cytokine responses, and variable disease severity in people living with HIV (PLWH) coinfected with dengue virus.

## 6 Diagnostic challenges and innovations

Dengue–HIV coinfections often go undiagnosed due to overlapping febrile syndromes. Misdiagnosis is common, particularly when dengue symptoms mimic acute retroviral syndrome. A Swiss case highlighted how dengue suspicion delayed the diagnosis of acute HIV infection, leading to irreversible optic neuropathy [19].

Encouragingly, rapid diagnostic tools like the OraQuick® HIV-1/2 test have maintained high specificity even in dengue-positive patients, proving useful for differential diagnosis in dengue-endemic areas [20]. RNA pooling techniques have also proven effective in detecting undiagnosed acute HIV cases during dengue outbreaks, especially in resource-limited settings [21].

Advanced biosensing technologies, including **graphene-based sensors** and **CRISPR-Cas systems**, have demonstrated remarkable potential for rapid, multiplexed dengue–HIV diagnostics in preclinical evaluations. These platforms offer high analytical sensitivity and portability, but **clinical validation remains limited**, and **standardized benchmarks** such as *limit of detection*, *specificity*, and *stability under field conditions* are yet to be established. Current prototypes have shown sensitivity comparable to RT-PCR in controlled settings, but **no large-scale clinical trials or regulatory approvals** have yet been completed [22,23]. Consequently, while these tools hold significant promise for point-of-care diagnostics, they should be viewed as **complementary to, rather than replacements for, validated molecular assays** until further evidence emerges. The diagnostic accuracy of dengue assays is influenced by the degree of immunosuppression and the timing of sample collection. In people living with HIV (PLWH), reduced humoral and cellular immune responses may result in delayed or attenuated antibody production, lowering the sensitivity of IgM- and IgG-based assays. The NS1 antigen test maintains relatively high specificity but its sensitivity declines after the fifth day of illness, while RT-PCR remains the gold standard during the early viremic phase. In advanced HIV infection, low CD4 counts and high viral loads have been associated with false-negative serologic results, emphasizing the importance of combining antigen and molecular tests where feasible. Diagnostic interpretation should therefore consider the immune status of the patient and the window periods of the respective tests. This distinction is critical in differentiating dengue from acute retroviral syndrome, where overlapping symptoms (fever, rash, lymphadenopathy, and myalgia) often lead to misdiagnosis in endemic regions [5,20,19].

## 7 Mathematical modeling and therapeutic development

Mathematical modeling has been used to simulate the co-dynamics of HIV and dengue. Fractional-order models incorporating various derivatives (Caputo, Caputo-Fabrizio, Atangana-Baleanu) have demonstrated the impact of controlling one virus on the spread of others [24]. These models support integrated disease control policies and highlight the need for synchronized surveillance systems.

On the therapeutic front, novel peptides derived from the DENV capsid protein, such as pepRF1, have demonstrated potent anti-HIV activity via CXCR4 antagonism in in vitro assays [25]. Although these findings suggest dual-benefit potential, pepRF1 remains in the preclinical stage, without animal or human trial validation. Therefore, its translation to clinical use will require rigorous pharmacokinetic, toxicity, and regulatory evaluation before integration into HIV–dengue therapeutic strategies.

## 8 Co-infections in sub Saharan Africa

Across sub-Saharan Africa, dengue transmission has expanded in urban and peri-urban areas, creating an under-recognized overlap with HIV care. In Abuja, Nigeria, 44.4 % of febrile people living with HIV (PLWH) on antiretroviral therapy were seropositive for dengue virus IgG, while 29.2 % had Plasmodium falciparum, indicating a high burden of co-infection and diagnostic overlap in febrile HIV cases [8]. Similarly, a multi-regional Nigerian serosurvey reported 44.7 % dengue-flavivirus IgG, 19.2 % Zika-flavivirus IgG, and 6.2 % dengue–Zika co-seropositivity, confirming widespread arboviral exposure that complicates the interpretation of febrile illnesses in HIV programs [26]. Because flavivirus serology is prone to cross-reactivity with other flaviviruses and prior yellow fever vaccination, diagnostic algorithms should emphasize NS1 antigen or RT-PCR testing for acute cases [26,27]. Clinical studies show no consistent increase in severe dengue among PLWH, although risk may be higher in those with advanced immunosuppression or secondary dengue infections [28]. Integration of dengue screening into HIV fever protocols, alongside malaria testing and vector-control interventions, is therefore critical for accurate case management in endemic African regions [[26], [27], [28]].

## 9 Broader coinfections and public health implications

Dengue coinfections do not exist in isolation. In many regions, HIV-positive individuals are also at risk for malaria, HBV, and chikungunya virus (CHIKV). One study in Nigeria found that nearly half of febrile HIV patients were coinfected with both dengue and malaria [8]. Similar interactions have been reported in Madagascar, where PLWH showed high seroprevalence to a range of arboviruses, including CHIKV and West Nile virus [10].

Autochthonous dengue outbreaks in Europe, such as the one in Italy in 2023, reflect how climate change and global mobility are facilitating dengue spread into non-endemic areas. Such scenarios demand updated public health strategies that include HIV-infected populations in vaccine trials and outbreak preparedness plans [7].

Integrating dengue surveillance with HIV care, improving diagnostic access, and fostering innovation in immunization strategies are key components for reducing the burden of these intertwined diseases [3,15]. Public health implications and reporting context. Evidence from Indonesia illustrates how reporting trends can shift with a mix of climate variability (Niño3.4/IOD), vector-control coverage, surveillance practices, and possible flavivirus cross-immunity: in 2017, notified dengue incidence fell ∼71 % (77.96 → 22.55 per 100,000) with only a slight CFR reduction (0.79 %→0.75 %), while national analyses found no clear correlation between incidence/CFR and larva-free index or integrated vector-management coverage; changes to surveillance definitions were also unlikely to explain the decline, and correlations with ENSO/IOD were suggestive but not statistically significant at annual resolution. These findings underscore that apparent declines can reflect multi-factor drivers (climate oscillations, heterogeneous control uptake, immunity cycles, and measurement effects), reinforcing the need in HIV programs to interpret dengue trends alongside diagnostic capacity (PCR/NS1 vs serology), case definitions, and subnational data quality, particularly in co-endemic African settings. Recent epidemiological analyses highlight that dengue reporting and case-fatality trends in endemic regions are shaped by surveillance definitions, vector control coverage, and climate variability, factors equally relevant to HIV program integration [29].

## 10 Limitations

This narrative review has several limitations that should be acknowledged. First, most of the available data on HIV–dengue coinfection come from cross-sectional or single-center studies, limiting the ability to establish temporal or causal relationships. Second, many seroprevalence reports rely solely on IgM or IgG assays without confirmatory plaque-reduction neutralization testing (PRNT), which may lead to overestimation of dengue exposure in flavivirus co-endemic regions. Third, regional data gaps persist across sub-Saharan Africa and parts of Latin America, where diagnostic capacity and routine dengue surveillance remain limited. Fourth, variability in immune status among people living with HIV—particularly those with low CD4 counts or incomplete viral suppression—may influence test performance and disease presentation, yet few studies stratify data by immunological markers. Finally, emerging diagnostic technologies such as graphene-based biosensors, CRISPR-Cas platforms, and peptide-based therapeutics (e.g., pepRF1) remain in preclinical or prototype stages without clinical validation or regulatory approval. These limitations highlight the need for well-designed, multicenter longitudinal studies and improved laboratory confirmation methods to refine our understanding of dengue virus dynamics in immunocompromised populations.

## 11 Conclusion

Dengue virus infection represents an evolving clinical and public health concern, particularly in regions where HIV is also endemic or emerging. Coinfection can produce atypical, blunted, or severe manifestations, influenced by immune status and viral interactions. The growing overlap with other febrile illnesses further complicates diagnosis, treatment, and surveillance.

Emerging diagnostic tools like graphene and CRISPR-based biosensors offer promising solutions for rapid, field-deployable detection. At the same time, advances in immunology and therapeutic development, including DENV-derived peptides, highlight the importance of cross-disciplinary innovation.

Ultimately, more longitudinal studies and integrated approaches are needed to define the natural history, optimize diagnostics, and develop therapeutic strategies tailored for dengue virus infections, especially among immunocompromised populations.

## Declaration of competing interest

The author declares that there are no known competing financial interests or personal relationships that could have appeared to influence the work reported in this paper.
